# Low-Loss Coupling of Quantum Cascade Lasers into Hollow-Core Waveguides with Single-Mode Output in the 3.7–7.6 μm Spectral Range

**DOI:** 10.3390/s16040533

**Published:** 2016-04-13

**Authors:** Pietro Patimisco, Angelo Sampaolo, Laura Mihai, Marilena Giglio, Jason Kriesel, Dan Sporea, Gaetano Scamarcio, Frank K. Tittel, Vincenzo Spagnolo

**Affiliations:** 1 Dipartimento Interateneo di Fisica, Università degli studi di Bari Aldo Moro e Politecnico di Bari, Via Amendola 173, Bari I-70126, Italy; pietro.patimisco@uniba.it (P.P.); angelo.sampaolo@uniba.it (A.S.); marilena.giglio@uniba.it (M.G.); gaetano.scamarcio@uniba.it (G.S.); 2 Department of Electrical and Computer Engineering, Rice University, 6100 Main Street, Houston, TX 77005, USA; fkt@rice.edu; 3 National Institute for Laser, Plasma and Radiation Physics Laser Metrology and Standardization Laboratory 409 Atomistilor, 077125 Magurele-Bucharest, Romania; laura.mihai@inflpr.ro (L.M.); dan.sporea@inflpr.ro (D.S.); 4 Opto-Knowledge Systems, Inc. (OKSI), 19805 Hamilton Ave., Torrance, CA 90502-1341, USA; jason@oksi.com

**Keywords:** single-mode output, hollow-core waveguide, quantum cascade laser, optical coupling

## Abstract

We demonstrated low-loss and single-mode laser beam delivery through hollow-core waveguides (HCWs) operating in the 3.7–7.6 μm spectral range. The employed HCWs have a circular cross section with a bore diameter of 200 μm and metallic/dielectric internal coatings deposited inside a glass capillary tube. The internal coatings have been produced to enhance the spectral response of the HCWs in the range 3.5–12 µm. We demonstrated Gaussian-like outputs throughout the 4.5–7.6 µm spectral range. A quasi single-mode output beam with only small beam distortions was achieved when the wavelength was reduced to 3.7 μm. With a 15-cm-long HCW and optimized coupling conditions, we measured coupling efficiencies of >88% and transmission losses of <1 dB in the investigated infrared spectral range.

## 1. Introduction

Many imaging and sensing applications require a good laser beam quality with circular symmetry, as close as possible to the fundamental transverse TEM_00_ mode, and essentially diffraction-limited low beam divergence with M^2^ values in the range 1.0–1.3 [1,2,3]. Commercial laser sources are often packaged with internal optics providing collimated output beams, which define and stabilize the output laser mode. These are typically designed and optimized to provide beam divergence of a few milliradians. However, the beam profile often does not result in a single-mode Gaussian-like shape, and typically exhibits an elliptical profile. Spatial defects, such as beam tails, can be easily removed by means of a pinhole. The most common beam collimating system is composed of an aspheric lens, which focuses the laser beam through a pinhole, and collimating optics, which collects the light passing through the pinhole [4,5]. If the laser beam contains higher-order modes, a modal filter for beam cleaning is essential. The ideal modal filter rejects all input field components that have no overlap with the fundamental mode of the filter, without attenuation of this mode [6]. Chalcogenide glass fibers are the most used single-mode solid-core optical waveguides in the infrared spectral range. They have a broad infrared wavelength transmission window between 1.5 and 11 μm. They are usually based on a step-index configuration and are designed to be single mode for wavelengths above a selected cut-off wavelength value. The coupling efficiency has a maximum at a specific wavelength and drops rapidly in the remaining operational spectral range. In the chalcogenide glass family, those based on As-S-Se exhibit single-mode delivery in the near infrared region (<5.5 µm) with and a minimum transmission loss of approximately 0.1 dB/m but suffering a coupling efficiency as low as 60%–70% [7]. For the infrared spectral range >5 μm, photonic crystal fibers have been used to provide single-mode delivery at 3.39 μm, 9.3 μm, and 10.6 μm [8], with losses around 2 dB/m at 10.6 μm [9].

Hollow-core waveguides (HCWs) can also act as efficient modal filters in the mid-IR range [10,11]. HCWs are optical fibers capable of single-mode mid-IR beam delivery [12,13] and are composed of a cylindrical glass capillary tube and metallic/dielectric layers deposited inside a hollow core. The spectral response of HCWs is determined by the thickness and the surface quality of the dielectric layer deposited inside the tube. The guided mode with lowest losses is a hybrid mode characterized by an optical power distribution corresponding to a Gaussian-like profile. Higher-order modes show losses that are larger than the fundamental one when traveling through the fiber. Hence, a minimum fiber length will exist that allows only the fundamental mode transmission through the fiber [12,13,14]. In this limit, the output beam exiting from the HCW will have an approximate Gaussian intensity distribution. Minimum loss condition and single-mode output beam can be achieved by optimizing the laser beam coupling into the HCW. This can be realized by using a lens that focuses the laser beam onto the HCW entrance. Experimentally, HCWs with bore diameters *d* ~ 30 times the wavelength have been shown to provide mid-IR laser beam delivery with low losses and single-mode output. [10]. In particular, HCWs with a bore size of *d* = 300 µm have been successfully employed in the range 7.6–11 µm for single-mode operation and optical losses of <1 dB/m have been measured [15,16,17]. Single-mode operation in the spectral range 5.1–10.5 μm was also demonstrated, employing HCWs with a bore size of 200 µm and internal layers specifically designed to reduce internal losses in the 5–12 µm spectral range [18,19].

In this work, we investigated the beam-guiding performances of HCWs fabricated by OptoKnoweldge Systems Inc. (OKSI) with a bore size of 200 µm and internal coatings realized to enhance the spectral response at 3.5 µm. To verify the possibility to achieve HCWs single-mode operation down to wavelengths shorter than 5 µm, we employed five quantum cascade lasers (QCLs) with emissions in the spectral range 3.7–7.6 μm and three HCWs with different lengths in the range 15–50 cm. We coupled the HCWs with the QCL sources by using appropriate focusing lenses. With optimized coupling conditions, single-mode and Gaussian-like outputs throughout the 4.5–7.6 µm range were observed, while quasi single-mode transmission, with only small spatial distortions, was achieved at 3.7 μm. In the last part of the manuscript, we compared the optical performance of a HCW with a bore size of 300 μm, with that of a HCW with a bore size of 200 μm, by coupling both with a 7.6 μm QCL beam. We demonstrated that, using a HCW with a 300 μm bore size, it is not possible to achieve single-mode operation at wavelength λ ≤ 7.6 μm [17].

## 2. Experimental Setup

**Figure 1:**
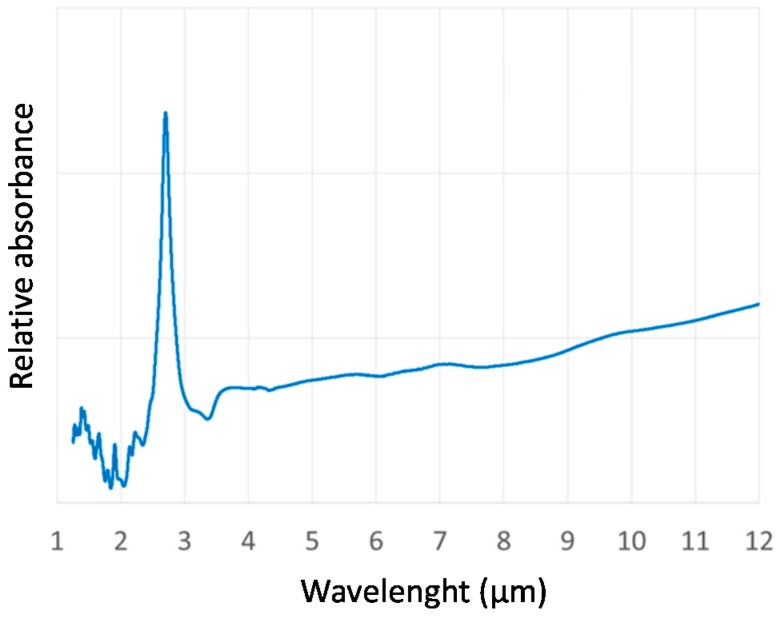
Hollow-core waveguide (HCW) relative absorbance measured in the range 1–12 μm using a FTIR spectrometer.

Three HCWs with a circular bore, having a cross-section diameter *d* = 200 μm and lengths of 15 cm, 30 cm, and 50 cm, produced by OKSI, were used in this work. Fabrication of the HCWs is accomplished using a wet chemistry process developed by Harrington *et al.* [12]. A silver (Ag) layer is deposited inside the glass capillary tube by flowing a silver solution through the tube using a peristaltic pump. After which, a dielectric layer is formed by flowing an iodine solution that reacts with the silver to form AgI. The longer the iodine solution flows, the thicker the resulting dielectric layer becomes. By controlling the thickness of the AgI dielectric layer, the transmission window of the fiber can be optimized for a specific wavelength range. For the HCWs employed in this work, both the reflective layer and the dielectric layer were optimized for shorter wavelengths than previously demonstrated for single-mode hollow fibers [18,19]. Modifications to the standard Ag coating technique were employed to produce a smoother surface and minimize scattering, which scales as 1/λ^2^. The AgI coating was designed to provide low propagation losses in the mid-infrared spectral range from 3.5 µm to 12 µm, as confirmed by the absorbance profile shown in Figure 1.

**Figure 2:**
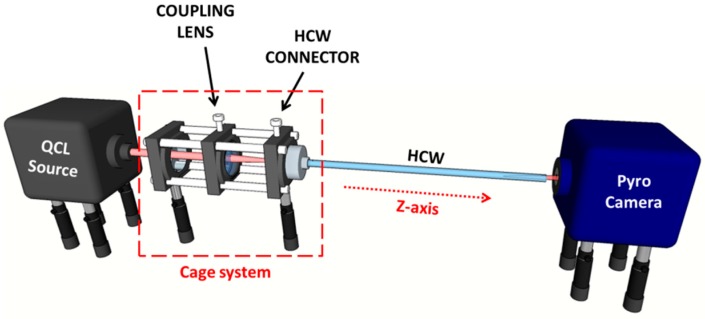
Schematic of the experimental setup used to optically couple quantum cascade laser (QCL) sources and the HCWs. A ZnSe lens is used to focus the collimated beam exiting from the QCL onto the waveguide entrance. An infrared pyrocamera detects the profile of the beam exiting from the HCW.

The optical coupling between the QCL and the HCW was carried out by employing the experimental setup schematically represented in Figure 2.

In order to investigate the beam guiding properties of HCWs in a wide mid-infrared spectral range, we employed four commercial tunable, external cavity QCLs (Pranalytica) operating at 3.7 μm (model MONOLUX 38), 4.5 μm (model MONOLUX 45), 4.9 μm (model MONOLUX 53), and 7.3 μm (model MONOLUX 74), respectively. All Monolux QCLs are equipped with custom-designed ZnSe aspherical collimating lenses, which are optimized to provide low output beam divergence. In order to realize fine adjustments of the optical coupling between the laser beam and the HCW, we developed a cage system, which includes a mount that allows a fine adjustment of the coupling lens position in the *x*–*y* planes and a fiber connector mounting to hold the HCW, as shown in Figure 2. The lens has a diameter of 1/2”, and the cage system allows fine adjustments to the distance between the lens and the HCW entrance along the optical *z*-axis. The HCW connector allows changes of the HCW position entrance with respect to the focused laser spot. With such an optomechanical system, both the azimuthal and polar angles of the waveguide entrance with respect to the laser beam direction can be precisely adjusted. The far field mode profile of the laser beam exiting the HCW was recorded by means of an infrared pyro-camera (Pyrocam III, Ophir Spiricon, 124 × 124 pixels, spatial resolution of 100 μm), positioned at a distance of about 2 cm from the HCW exit. The optical power of the laser beam exiting from the HCW was measured by replacing the pyrocamera with an infrared power meter detector.

## 3. Optical Coupling Conditions

The coupling efficiency of a fiber can be calculated from the overlap integrals between the guided modes and the laser beam [20]. The intensity distribution profile of the laser beam at the waveguide entrance can be approximated by two-dimensional (2D), Gaussian elliptical function G(*x*,*y*) given by:

(1) $$G(x,y)=\frac{1}{2\pi \sigma_{0,x}\sigma_{0,y}}e^{-\frac{x^{2}}{2\sigma_{0,x}^{2}}}e^{-\frac{y^{2}}{2\sigma_{0,y}^{2}}}$$

where (*x_0_, y_0_*) are the spatial coordinates of the center, and σ*_0,x_* and σ*_0,y_* are the *x* and *y* spreads at the waveguide entrance, respectively. When the 2D laser beam enters the fiber, it transforms into a composition of the fiber modes. Hence, a coupling efficiency can be calculated for each *n*-th guided mode as:

(2) $$\eta_{n}=\frac{{\left[\int_{-\infty }^{+\infty }\int_{-\infty }^{+\infty }G(x,y)E_{n}(x.y)dxdy\right]}^{2}}{\int_{-\infty }^{+\infty }\int_{-\infty }^{+\infty }G^{2}(x,y)dxdy\int_{-\infty }^{+\infty }\int_{-\infty }^{+\infty }E_{n}{}^{2}(x.y)dxdy}$$

where *E_n_(x,y)* is the *n*-th waveguide mode represented by a Bessel function. The lowest-order guided mode *E_1_(x,y)* has a Gaussian spatial profile with circular symmetry. As stated previously, to achieve the optimum coupling conditions, as much of the available laser beam power as possible must be coupled in the lowest-order mode. Higher-order modes suffer increased losses, and the power coupled into these modes is rapidly dissipated as the beam propagates through the waveguide. If the HWC fiber is long enough, only the *E_1_(x,y)* mode survives, and a single-mode output at the waveguide exit is obtained [20]. Numerical calculations have shown that coupling efficiencies η*_1_* higher than 0.89 can be obtained when both the ratios *w_0,x_/d* and *w_0_,_y_/d* are in the 0.5–0.75 range, where *w_0,x_ = 2*σ*_0,x_* and *w_0,y_ = 2*σ*_0,y_* are the beam diameters at the waveguide entrance. In our case, this implies that the laser beam at the waveguide entrance must have both *w_0,x_* and *w_0,y_* in the range 100–150 μm. Hence, a coupling lens with an adequate focal length must be selected to achieve this condition. Measurements of beam diameters as small as 100 μm cannot be resolved by available infrared cameras. Therefore, the beam widths *w_0,x_* and *w_0,y_* of the focused laser beam at the waveguide entrance was estimated by measuring the diameters *w_x_* and *w_y_* of the laser beam at the coupling lens using the following expression:

(3) $$w_{0,i}=\frac{w_{i}}{\sqrt{1+{\left(\frac{\pi w_{i}^{2}}{\lambda R}\right)}^{2}}},\text{ with }i=x,y$$

where *R* is the radius of curvature of the coupling lens. Starting from the laser beam profiles on the lens, the beam diameters *w_x_* and *w_y_* can be extracted by evaluating the second-order moments of the beam intensity distribution, as reported in [11].

## 4. Beam Profiles and Related Spatial Quality

**Figure 3:**
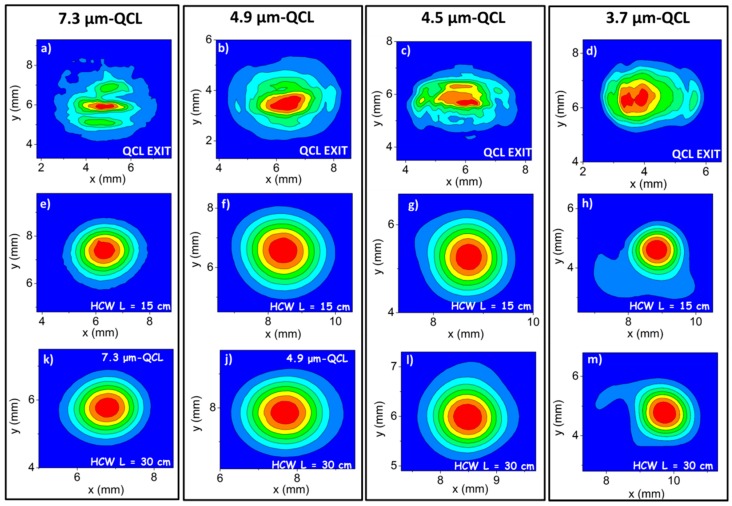
(**a**–**d**) Beam profiles of the QCL at 7.3 μm (**a**), at 4.9 μm (**b**), at 4.5 μm (**c**), and at 3.7 μm (**d**), recorded by positioning the infrared pyro-camera 7 cm away from the QCL exit. (**e**–**m**) The HCW output beam profiles were measured for the 15-cm- (**e**–**h**) and 30-cm-long (**k**–**m**) waveguides. The focusing lens was positioned at a distance of 7 cm from the QCL output, and the pyrocamera was located at ~2 cm from the HCW exit.

The QCLs beam profiles were recorded by positioning the infrared pyro-camera 7 cm away from the QCL exit and are shown in Figure 3a–d. In Table 1, we report both the measured *w_x_* and *w_y_* beam diameters and the calculated *w_0,x_* and *w_0,y_* values obtained using Equation (3), based on the coupling lens selected for each QCL source.

For our experiments, a lens with focal length *f* = 50 mm was selected for the QCL operating at 7.3 µm and *f* = 75 mm for the other three QCLs, since these *f* values provide *w_0,x_* and *w_0,y_* dimensions falling in the range 96–144 µm, nearly identical to the optimal one, as described above. We coupled the QCL beams into the waveguide by positioning the focusing lens 7 cm from the QCL output. The best coupling conditions were obtained by maximizing the HCW output power. Figure 3e–m show the beam profiles obtained at the exit of a 15-cm- and 30-cm-long HCWs in straight positions for the QCL emitting at 7.3 μm (Figure 3e,k), at 4.9 μm (Figure 3f,j), 4.5 μm (Figure 3g,l), and 3.7 μm (Figure 3h,m). To collect the beam profile, we positioned the pyro-camera ~2 cm far from the waveguide exit. 

The measured profiles demonstrated that, despite the low quality of the input laser beam, HCWs with a core size as small as 200 μm allow single-mode output with a Gaussian-like beam profile down to a wavelength of 4.5 μm. Indeed, the QCLs mode are perfectly matched to the *E_1_(x,y)* guided mode, as shown by the circular-symmetric beam output [21]. Similar single-mode results were obtained for the 50-cm-long HCW. For the shorter wavelength QCL emitting at 3.7 μm, the acquired profiles show a Gaussian-like pattern with small tails. However, only <3% of the total power is contained in the beam tails, which can be removed using a pinhole. A similar profile was also observed for the 50-cm-long fiber.

The HCWs output beam spatial quality can be quantified by calculating the parameter M^2^ which compares the laser beam angular divergence in the two transverse directions with a diffraction-limited Gaussian beam, which has M^2^ = 1. For the beam exiting from the QCLs, the M^2^ value can be defined as:

(4) $$M_{i,QCL}^{2}=\frac{\theta_{i,QCL}}{2}\frac{\pi w_{i}}{\lambda },\text{ with }i=x,y$$

where λ*/(*π *w_i_)* is the beam divergence half-angle of a diffraction-limited Gaussian beam, and θ*_x(QCL)_* and θ*_y(QCL)_* are the divergence half-angles of the QCL beams. The M^2^ value of the beam exiting from the HCWs can be defined as the ratio between the half-angle beam divergence of the beam and the theoretical half-angle beam divergence:

(5) $$M_{i,HCW}^{2}=\frac{\theta_{i,HCW}}{2}\frac{\pi d}{2.4048\cdot \lambda },\text{ with }i=x,y$$

where 2.4048∙λ/(π·d) is the theoretical half-angle divergence, calculated by assuming that only the *E_1_(x,y)* guided mode exits from the waveguide. For both, the QCLs and the waveguide outputs, half-angle beam divergences of the real beam can be calculated by extracting the beam widths *w_x_* and *w_y_* from the beam profiles acquired at different distances using the experimental approach reported in [22]. Results obtained for the output of all four QCLs together with results for the same beams after exiting the 15-cm-long HCW are listed in Table 2.

The calculated M^2^ values confirm that the laser beam exiting from the *L* = 15 cm HCW shows a substantial improvement in spatial quality with respect to the input laser beam. Similar M^2^ values were obtained for the 30-cm- and 50-cm-long HCWs. 

## 5. Total Losses and Coupling Efficiency

The propagation losses *Lp* of an HCW can be calculated by using the following expression:

(6) $$L_{p}(dB)=-10\log_{10}\left(\sum_{n}\eta_{n}e^{-2\alpha_{n}L}\right)$$

where α*_n_* are the attenuation coefficients related to the *n*-th guided modes. These coefficients depend on the laser wavelength and the optical properties of the dielectric and metallic layers deposited inside the HCW. For their estimation, we used the relation provided by Miyagi and Kawakami [23]:

(7) $$\alpha_{n}={\left(\frac{u_{n}}{2\pi }\right)}^{2}\frac{8\lambda^{2}}{d^{3}}\left(\frac{n_{m}}{n_{m}{}^{2}-k_{m}{}^{2}}\right)\left\{\frac{1}{2}{\left[1+\frac{n_{d}^{2}}{{\left(n_{d}^{2}-1\right)}^{1/2}}\right]}^{2}\right\}$$

where *n_m_* and *k_m_* are the real and imaginary parts of the complex index of the silver layer, *n_d_* is the complex index of the silver iodine layer, and *u_n_* is the *n*-th root of the zero-order Bessel function. The values of *n_m_*, *k_m_*, and *n_d_* were calculated by using relations reported in [24]. Experimental values of the HCW total losses can be calculated from the ratio between the power at the waveguide entrance *I_0_* and that measured at the fiber exit *I_S_* as:

(8) $$Losses(dB)=10\log_{10}\left(\frac{I_{0}}{I_{S}}\right)$$

**Figure 4:**
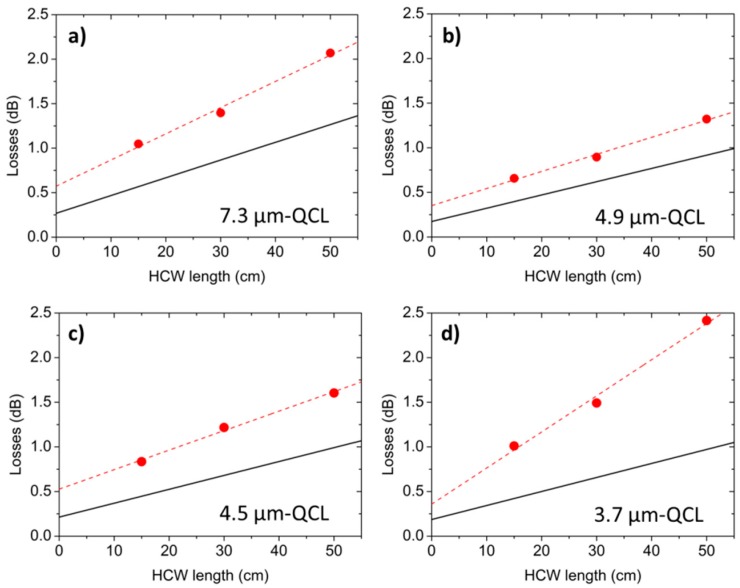
(**a**–**d**) Total losses (●) measured when coupling the 7.3-μm- (**a**), 4.9-μm- (**b**), 4.5-μm- (**c**) and 3.7-μm-QCL (**d**) beams into the HCWs having lengths of 15, 30, and 50 cm, plotted as a function of the HCW length. Dashed lines are linear fits to the data. Solid lines are the theoretical losses calculated by using Equations (6) and (7).

Figure 4a–d show the total losses measured for the 15-cm-, 30-cm- and 50-cm-long HCWs, using QCLs emitting at 7.3 μm (Figure 4a), 4.9 μm (Figure 4b), 4.5 μm (Figure 4c), and 3.7 μm (Figure 4d), for their respective best coupling conditions. Figure 4 also includes the theoretical losses, calculated by using Equations (6) and (7), as a function of the HCWs length and assuming that the waveguide modes *E_n_(x,y)*, with n ranging from 1 to 5, propagate inside the waveguide.

Measured optical losses are higher than the calculated ones. These discrepancies can be attributed to the poor spatial laser beam quality, which impacts: (i) the estimation of theoretical coupling efficiencies (calculated by assuming a perfect two-dimensional Gaussian elliptical profile for the laser beam) and the consequent propagation losses; and (ii) the beam waist sizes at the waveguide entrance. From the intercept of the linear fit in Figure 4, we extracted the coupling efficiency values η*_exp_*, reported in Table 3, together with the theoretical values calculated by using Equations (1) and (2). A very good data agreement (<7% discrepancy) was obtained.

**Figure 5:**
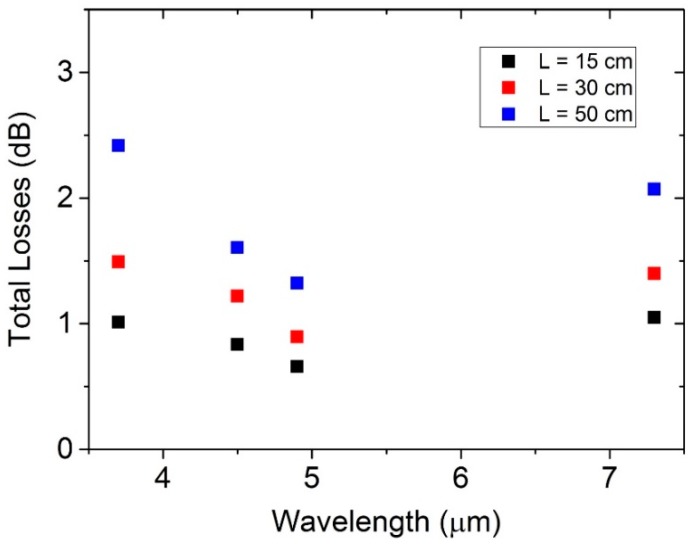
(**a**–**d**) Total losses as a function of the QCLs emitting wavelengths for HCWs with L = 15 cm (▀), 30 cm (▀), and 50 cm (▀).

In Figure 5, we report the total losses, plotted as a function of the lasers wavelength for all three HCW lengths. 

This graph clearly shows that, for all investigated HCW lengths, the measured loss changes are less than 1 dB when the laser wavelengths span from 3.7 to 7.3 μm, confirming that the internal coatings enhance the HCW spectral response to 3.7 µm. Moreover, the losses remain ≤1 dB in the investigated wavelength range for the 15-cm-long HCW.

## 6. Influence of Bore Diameter and HCW Length on the Output Beam Quality

**Figure 6:**
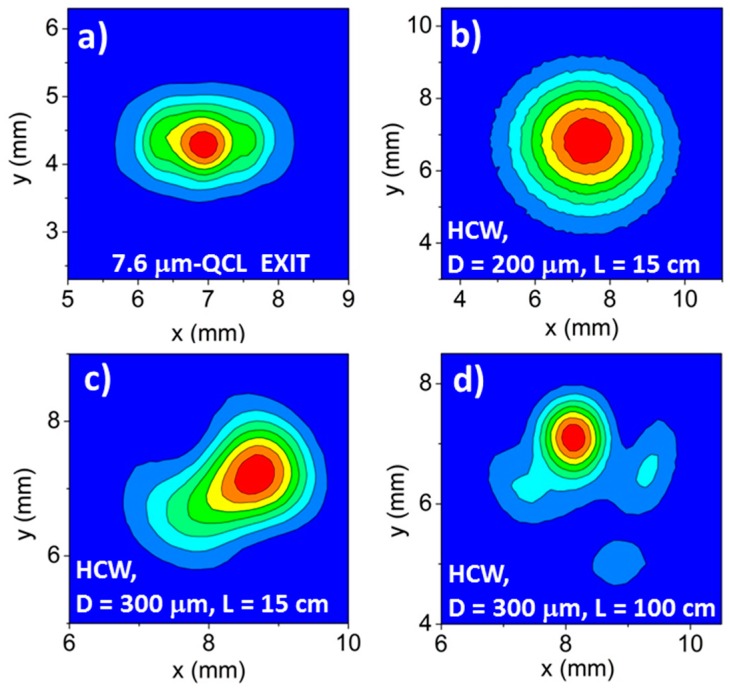
(**a**) Beam profile at the exit of DLS-QCL emitting at 7.6 μm. (**b**–**d**) Beam profiles with a 200-μm-core-sized and 15-cm-long HCW (**b**); 300-μm-core-sized and 15-cm-long HCW (**c**); and 300-μm-core-sized and 100-cm-long HCW (**d**) exits. The focusing lens was positioned at a distance of 5 cm from the QCL output and the pyrocamera was located at ~2 cm from the HCWs exit.

The *1/d^3^* dependence (Equation (7)) predicts a strong increase in HCW losses as the fiber bore diameter is reduced. A large bore diameter cannot ensure a single-mode profile at the waveguide output. From Equation (6), we can see that for large bore sizes α*_n_* is small and higher-order modes have lower losses with respect to those obtained for small bore sizes, for which α*_n_* is larger. For example, calculations of attenuation coefficients using Equations (7) and (8) at λ = 7.6 μm and *L* = 1 m show that, for *d* = 200 μm, losses related to the second-order mode (*n* = 2) are 11.3 dB, but decreases to 3.3 dB when *d* is 300 μm. Thus, there is significantly less mode mixing at the HCW output in waveguides with reduced bore size. Mode mixing can be reduced by increasing the fiber length at the expense of larger optical losses. The spatial quality of the input laser beam must also be taken into account, since it influences the input power percentage coupled into the guided modes (see Equation (2)). The larger the amount of input power coupled into the high-order modes, the larger their contributions to total propagation losses will be. As a result, a HCW with a small bore size can exhibit a single-mode output profile, but with optical losses significantly higher than those theoretically predicted. To investigate the influence of the HCW bore size on optical losses and output beam profile, we coupled a 200-µm- and 300-µm-core-sized HCWs, both having a length of 15 cm, with a Daylight Solution external cavity QCL (DLS-QCL) emitting at 7.6 μm. The beam profile at the DLS-QCL exit is shown in Figure 6a, characterized by *w_x_ =* 2.56 mm and *w_y_ =* 1.79 mm. The DLS-QCL was coupled with a 200-µm-core-sized HCW by using a coupling lens with a focal length of 25 mm (resulting in *w_0,x_/d* = 0.48 and *w_0,y_/d* = 0.67). For the 300-µm-core-sized HCW, a focal length of 50 mm (*w_0,x_/d* = 0.63 and *w_0,y_/d* = 0.88) was used. The output profiles of the 200-μm- and 300-μm-core-sized HCWs (both with *L* = 15 cm) are shown in Figure 6b,c, respectively.

The intensity distribution at the output of the 200-μm-core-sized and 15-cm-long HCW, shown in Figure 6b, is single mode. At the HCW exit, we measured optical losses of 0.74 dB, slightly higher than those theoretically predicted (0.65 dB) by using Equations (6) and (7). This confirms that the quality of the laser beam influences the total losses, but not the output beam profile. Conversely, the intensity distribution is multimodal for the 300-μm-core-sized and 15-cm-long HCW, as shown in Figure 6c. We measured losses as high as 1.14 dB, 0.56 dB higher than the theoretical value. This discrepancy is largely due to the propagation of higher-order modes, which have greater losses than the lowest-order *E_1_* mode. To verify whether the contribution to higher-order modes at the 300-μm-core-sized HCW exit can be suppressed by increasing the fiber length, we coupled the DLS-QCL with a 100-cm-long, 300-µm-core-sized HCW. The coupling conditions were the same as used for 300-μm-core-sized HCW. The acquired output beam profile is reported in Figure 6d. Clearly, the profile is still multimodal, with optical losses of 3.45 dB (1.62 dB are the theoretical losses), demonstrating that the only way to achieve single-mode beam delivery in the spectral range of 4.5–7.6 μm is to use HCWs with a bore size as small as 200 μm. For this condition, a length as short as 15 cm is sufficient to provide a single-mode output, even if the spatial quality of the input laser beam is not good. For longer fibers, single-mode output is ensured but optical losses increase. For the spectral range 8–11 μm, both 200-μm and 300-μm diameters can guarantee single-mode beam delivery [16,17,18], but it is preferable to employ a bore size of 300 μm, since it provides lower optical losses.

## 7. Conclusions

Prior to this work, single-mode laser beam delivery in the mid-infrared range was demonstrated at λ ≤ 5.5 μm with solid core fibers [7,25] and at λ ≥ 5.1 μm by using HCWs [18]. In this work, despite the limited beam quality of the input laser beams, we demonstrated single-mode propagation down to 4.5 µm and nearly single-mode at 3.7 µm with only slight spatial distortions through HCWs with bore diameters of 200 μm. Thus, it was shown that there are single-mode fiber options covering the 3.7 µm–12 µm mid-IR spectral range. For the 15-cm-long HCW, we measured straight optical losses of ≤1 dB and a coupling efficiency of ≥88% in the investigated 3.7–7.3 µm spectral range. In all cases, the measured losses are higher than those theoretically predicted, and the discrepancies can be attributed to the limited quality of the input QCL beams. We also demonstrated that, using HCWs with a bore diameter of 300 µm, it is not possible to achieve single-mode operation for λ ≤ 7.6 µm (40 times lower than the bore size). Otherwise, 200-μm-core HCWs are capable of operating in single-mode down to wavelength 50 times lower than their bore sizes, which, to date, represents the largest *d/*λ ratio ever observed for HCWs.

## Figures and Tables

**Table 1:** *w_x_* and *w_y_* values calculated by using the second-order moments method; *w_0,x_* and *w_o,y_* values calculated from Equation (3) when a coupling lens with a focal length *f* is employed.

	7.3-μm QCL	4.9-μm QCL	4.5-μm QCL	3.7-μm QCL
*w_x_* (mm)	5.26	4.24	4.19	4.27
*w_y_* (mm)	4.79	3.97	3.48	3.67
*f (*mm*)*	50	75	75	75
*w_0,x_* (μm)	132	114	96	100
*w_0,y_* (μm)	144	122	120	116

**Table 2:** Divergence angles and M^2^ values for four QCLs and the 15-cm-long HCW, in two directions (*x* and *y*) orthogonal to the QCL beam propagating *z*-direction.

	7.3-μm QCL	4.9-μm QCL	4.5-μm QCL	3.7-μm QCL
θ*_x,QCL_* (mrad)	2.1	1.9	1.5	1.3
θ*_y,QCL_* (mrad)	2.0	1.7	2.2	1.1
*M^2^_x, QCL_*	4.75	5.16	4.39	4.71
*M^2^_y, QCL_*	4.12	4.32	4.85	3.42
θ*_x,HCW_* (mrad)	30.72	22.48	21.97	20.48
θ*_y,HCW_* (mrad)	31.53	22.22	22.30	19.92
*M^2^_x, HCW_*	1.12	1.18	1.26	1.44
*M^2^_y, HCW_*	1.15	1.17	1.28	1.40

**Table 3:** Theoretical coupling efficiencies into the lowest-order mode η_1_ and experimental η_ext_ values.

	7.3-μm QCL	4.9-μm QCL	4.5-μm QCL	3.7-μm QCL
η*_1_*	0.94	0.96	0.95	0.96
η*_exp_*	0.88	0.92	0.89	0.92
